# Bivalent Metal-Chelating Properties of Harzianic Acid Produced by *Trichoderma pleuroticola* Associated to the Gastropod *Melarhaphe neritoides*

**DOI:** 10.3390/molecules25092147

**Published:** 2020-05-04

**Authors:** Gaetano De Tommaso, Maria Michela Salvatore, Rosario Nicoletti, Marina DellaGreca, Francesco Vinale, Assunta Bottiglieri, Alessia Staropoli, Francesco Salvatore, Matteo Lorito, Mauro Iuliano, Anna Andolfi

**Affiliations:** 1 Department of Chemical Sciences, University of Naples Federico II, 80126 Naples, Italy; gaetano.detommaso@unina.it (G.D.T.); mariamichela.salvatore@unina.it (M.M.S.); dellagre@unina.it (M.D.); frsalvat@unina.it (F.S.); 2 Council for Agricultural Research and Economics, Research Centre for Olive, Fruit and Citrus Crops, 81100 Caserta, Italy; rosario.nicoletti@crea.gov.it; 3 Department of Agricultural Sciences, University of Naples Federico II, 80055 Portici, Italy; assunta_bottiglieri@libero.it (A.B.); alessia.staropoli@unina.it (A.S.); matteo.lorito@unina.it (M.L.); 4 Department of Veterinary Medicine and Animal Productions, University of Naples Federico II, 80137 Naples, Italy; frvinale@unina.it; 5 Institute for Sustainable Plant Protection, National Research Council, 80055 Portici, Italy

**Keywords:** *Trichoderma* secondary metabolites, bioactive products, chelating metal, tetramic acids, harzianic acid complexes

## Abstract

Harzianic acid is a secondary metabolite of *Trichoderma*, structurally belonging to the dienyltetramic acid subgroup of the tetramic acids. Biological activities of harzianic acid are of great interest for its antimicrobial and plant growth-promoting activities, which might be related to its chelating properties. In the present work harzianic acid, isolated from cultures of a strain of *Trichoderma pleuroticola* associated to the gastropod *Melarhaphe neritoides*, was studied as a complexant agent of a number of biologically relevant transition metals (i.e., Zn^2+^, Fe^2+^, Cu^2+^, and Mn^2+^), using UV-VIS, potentiometry, MS and NMR techniques. Our findings show the coordination capacity of harzianic acid toward the above cations through the formation of neutral or charged complexes in a variable ratio depending on the metal and pH conditions.

## 1. Introduction

Species of *Trichoderma* (Sordariomycetes, Hypocreaceae) are widespread in every natural environment on earth, in connection with their extraordinary adaptive capacity to different ecological conditions and lifestyles [1,2]. Particularly, these fungi are able to establish various interactions with plants and other microbes [1,2,3,4]. At least in part, this is related to the production of secondary metabolites, belonging to several class of compound such as butenolides, epipolythiodioxopiperazines, thiosilvatins, pyrones, sorbicillinoids, terpenoids [5,6,7,8,9,10,11].

Tetramic acids represent a group of *Trichoderma* metabolites, including trichosetin [12] and harzianic acid and its analogs belonging to the subgroup of the dienoyltetramic acid according to a recent classification [13]. Harzianic acid, was first isolated from *Trichoderma harzianum* [14] and characterizes the chemotaxonomic profile of this species [15], along with its analogues isoharzianic acid (C-5′ epimer) [16], demethylharzianic acid, and homoharzianic acid [17]. The production of a new derivative harziaphilic acid was also detected in co-cultures with a strain of *Talaromyces pinophilus* [18]. However, after the spread of DNA sequencing in fungal taxonomy, recent revisions have shown *T. harzianum* to be a species complex made of many biological species [19,20], suggesting a more accurate assessment of the harzianic acid producers.

The absolute configuration *S*,*S* at the asymmetric carbons C-5′ and C-7 of harzianic acid was assigned by X-ray diffraction studies [21]. Moreover, harzianic acid and its stereoisomers were also prepared in six steps with an overall yield of 22% from the masked 4,4-disubstituted glutamic acid and a polyene fragment [22].

Recent studies reported intriguing bioactivities of harzianic acid, such as antimicrobial activity against phytopathogenic species (*Pythium irregulare*, *Sclerotinia sclerotiorum,* and *Rhizoctonia solani*), promotion of plant growth [21]. Some of these activities might be related to the chelating properties of this metabolite. In fact, it is documented that harzianic acid inhibits the serine/threonine phosphatase type 2A (PP2A) only in presence of zinc in a complex ligand-Zn^2+^ 2:1 [17]. Moreover, its complex with Fe^3+^ has been demonstrated and could be related to the growth promotion activity on tomato seedlings [23]. Hence, the improvement of knowledge on chelating properties of harzianic acid is of great interest to better understand its biological activities also against other microbial targets (i.e., virus).

Divalent metals (i.e., Mn, Fe, Co, Ni, Cu, and Zn) are essential micronutrients for all life forms, in particular for their catalytic activities, substrate stabilization, and as reaction intermediates. The detection of divalent metals is of special interest because their beneficial effects are affected by concentration. In this respect, many organisms produce metabolites with high metal-binding proprieties [24], and their interactions might be influenced by the capacity to regulate metal levels [25].

The aim of this work is to study the chelating properties of harzianic acid, isolated from a marine strain of *T. pleuroticola*, to evaluate the formation and stability of complexes formed with transition metals with biologically relevant functions (i.e., Cu^+2^, Fe^2+^, Mn^2+^, and Zn^2+^).

## 2. Results and Discussion

### 2.1. Identification of Strain L1 of Trichoderma pleuroticola Isolated from Melarhaphe neritoides

Strain L1 recovered from the gastropod *Melarhaphe neritoides* was identified according to morphological and molecular methods. A blast in GenBank of the TEF sequence obtained (Figure S1) yielded a strain of *T. pleuroticola* (T1295), commonly used as a reference in phylogenetic assessments involving this species [26], as the closest match, with an identity of 98.69% (query cover 100%). *T. pleuroticola* was separated from *T. harzianum* based on mycoparasitic strains from *Pleurotus* [27], but afterwards recovered from various contexts, including marine sediments [28].

### 2.2. Isolation and Identification of Harzianic Acid

**Figure 1:**
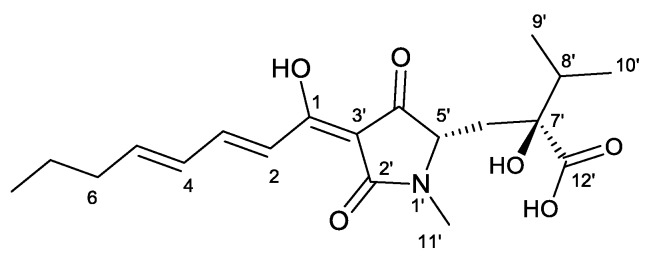
Structure of harzianic acid.

**Figure 2:**
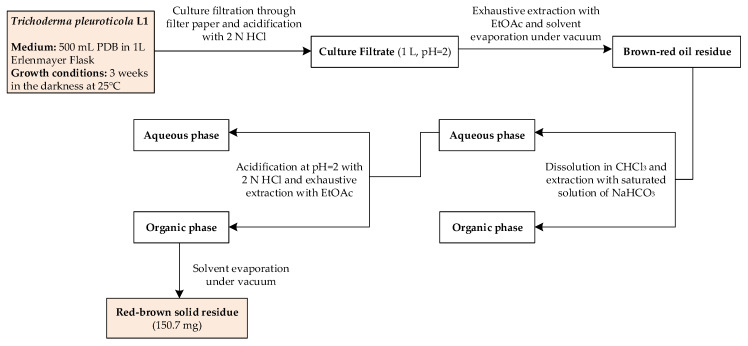
Schematic representation of harzianic acid extraction from *Trichoderma pleuroticola* L1 culture filtrate.

The metabolomic analysis (LC-MS approach) of L1 culture filtrates showed the presence of harzianic acid (Figure 1) as the major compound. LC-ESI mass spectrum, recorded in positive mode, exhibits peaks at *m*/*z* 366, 388 and 753 corresponding respectively to [M + H]^+^, [M + Na]^+^, [2M + Na]^+^ ions (Figure S2). In order to extract harzianic acid, culture filtrate was treated as previously described [16], with slight modifications. Particularly, a saturated NaHCO_3_ solution was used in order to separate neutral and basic substances, which are eventually contained in the crude extract. Harzianic acid was subsequently extracted with EtOAc following acidification of the basic aqueous phase (Figure 2). The NMR data, recorded in CDCl_3_ and CD_3_OD (Figures S3–S5), and optical rotation of the purified compound were in agreement with previous reports [22] (Table S1). Repeated chromatographic and spectral analysis did not demonstrate evidence of the presence of isomeric or degradation products of harzianic acid.

### 2.3. Determination of Protonation Constants of Harzianic Acid

**Figure 3:**
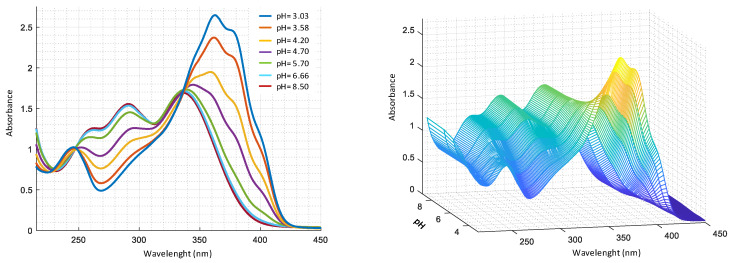
UV-VIS spectra as a function of pH for a solution 2.0 × 10^−4^ M harzianic acid in CH_3_OH/0.1 M NaClO_4_ (50:50 *w*/*w*) recorded at pHs: 3.03, 3.58, 4.20, 4.70, 5.70, 6.66, 8.50 (**left** 2D–**right** 3D graphics).

The experimental data were collected by both spectrophotometric titrations in UV-VIS region and potentiometric technique, where the pH measurements were conducted with glass electrode. By reason of low solubility of harzianic acid in aqueous solutions, the measures were performed in CH_3_OH/0.1 M NaClO_4_ 50:50 (*w*/*w*) as ionic medium. UV-VIS spectra relating to titration are reported in Figure 3.

By analysis of UV-Vis spectra (Figure 3), in acid solution, it is evident the presence of two bands at 350 nm and 250 nm, that shift in alkaline solution. Two isosbestic points are also observed, at 350 nm and 240 nm respectively, compatible with the presence of two acid–base equilibria as reported in Table 1. To assign the corresponding type of molecular transition to the absorption bands, spectra were recorded in solvents with different polarity, such as methanol and 2–propanol. In particular, at pH = 5.70, band at 250 nm in methanol shows a bathochromic shift (log ε=3.89), compared to 240 nm (log ε=4.10) in 2–propanol (Figure S6). This behavior can be associated with transitions involving the diene system orbitals. Furthermore, band at around 350 nm undergoes a hypsochromic shift −345 nm in methanol (log ε=4.12), compared to 360 nm (log ε=4.43) in 2–propanol, which can be assigned to transitions that involve the non-bonding electrons of oxygen in C4’ position [29]. On the basis of the experimental data obtained with potentiometric and spectrophotometric measurements and considering pKa of α-hydroxyacids [30], the protolytic constants of carboxylic and dienoyl groups were assigned as reported in Table 1.

**Figure 4:**
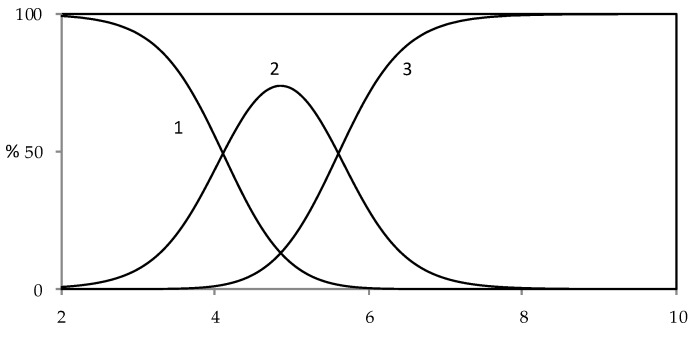
Distribution diagram of protolytic species for harzianic acid in CH_3_OH/0.1 M NaClO_4_ (50:50 *w*/*w*) (1: H_2_L; 2: HL^−^; 3: L^2^^−^).

By the acid constants, it is possible to build up the distribution curves for all the species present in this system, as reported in Figure 4.

**Figure 5:**
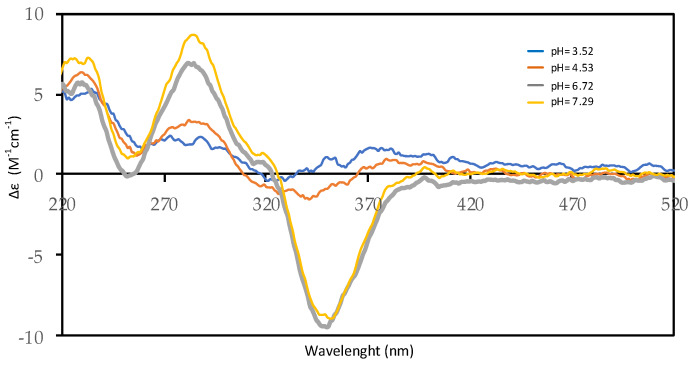
Far–UV CD spectra of 2.0 × 10^−4^ M harzianic acid in CH_3_OH/0.1 M NaClO_4_ (50:50 *w*/*w*) at different pH values (3.52, 4.53, 6.72, 7.29) (optical path 0.2 cm).

Circular dichroism measurements, conducted in the same conditions as potentiometric and spectrophotometric assessments, show, as the pH of the solution increases, the presence of two peaks (one positive Cotton effect at 285 nm and another negative at 350 nm) as reported in Figure 5. This behavior indicates an increase in the asymmetry of the molecule in alkaline solution, which affects the chirality in position C5′. In effect, by a keto–enolic equilibrium, that involves the carbonyl group in C4′, it is possible to invert the configuration in C5′.

### 2.4. Study of Complexation of Harzianic Acid with Metal Cations

**Figure 6:**
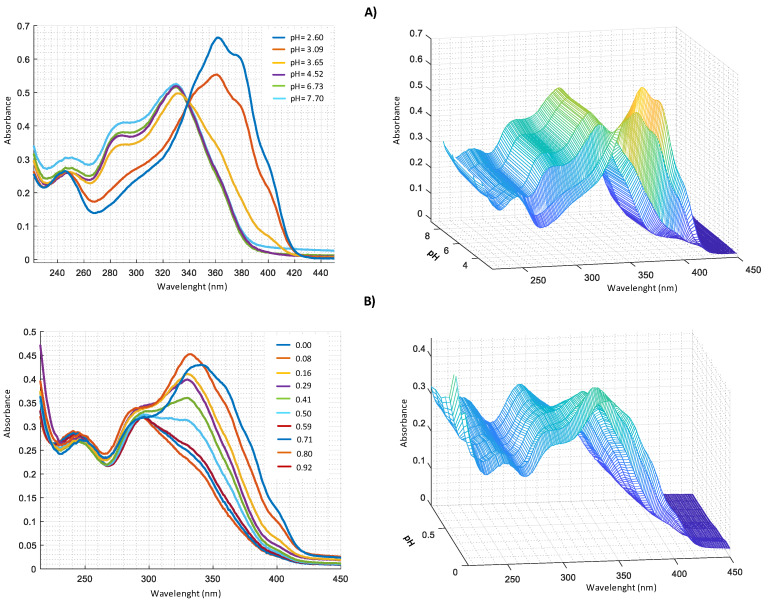
UV–VIS spectra in CH_3_OH/0.1 M NaClO_4_ (50:50 *w*/*w* of: (**A**) 2.0 × 10^−4^ M harzianic acid and 2.0 × 10^−4^ M Zn(ClO_4_)_2_ at different pH (2.60; 3.09; 3.65; 4.52; 6.73; 7.70); (**B**) at different metal/harzianic acid molar ratio (0.08; 0.16; 0.29; 0.41; 0.50; 0.59; 0.71; 0.80; 0.92) at pH = 6.50 (**left** 2D–**right** 3D graphics).

UV-VIS measurements carried out for Zn^2+^–harzianic acid system at different pH (Figure 6A) show the presence, in alkaline solution, of three bands at 251 nm (*log* ɛ = 3.45), 290 nm (*log* ɛ = 3.58) and 330 nm (*log* ɛ = 3.69). From spectra recorded at different metal/harzianic acid molar ratio (Figure 6B) (at fixed pH), two peaks at 335 nm (*log* ɛ = 4.15) and 294 nm (*log* ɛ = 3.99) are observed. Measurements show a considerable spectral variation up to a value of 0.5 metal/ligand molar ratio that is compatible with the formation of a 1:2 stoichiometric complex.

**Figure 7:**
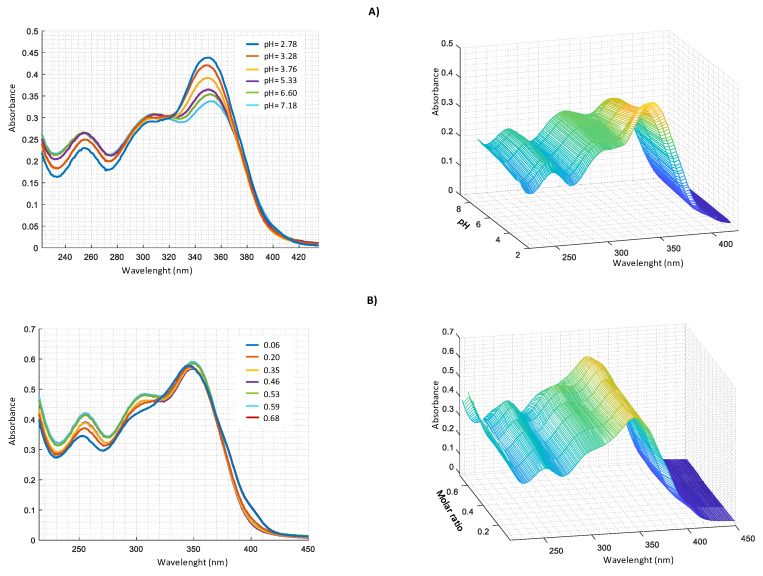
UV–VIS spectra in CH_3_OH/0.1 M NaClO_4_ (50:50 *w*/*w*) of: (**A**) 2.0 × 10^4^ M harzianic acid and 2.0 × 10^4^ M Cu(ClO_4_)_2_ at different pH (2.78, 3.28, 3.76, 5.33, 6.60, 7.18); (**B**) at different metal/harzianic acid molar ratio (0.06, 0.20, 0.35, 0.46, 0.53, 0.59, 0.68) at pH = 6.50 (**left** 2D–**right** 3D graphics).

**Figure 8:**
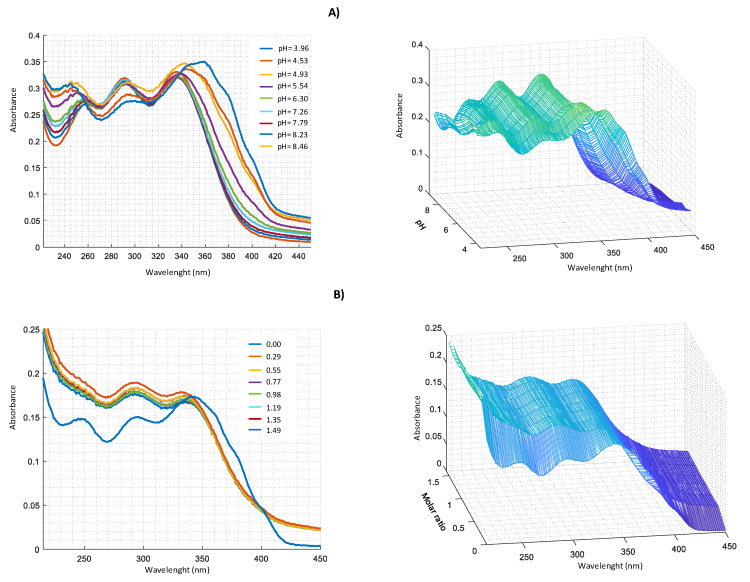
UV–VIS spectra in CH_3_OH/0.1 M NaClO_4_ (50:50 *w*/*w*) of: (**A**) 2.0 × 10^−^^4^ M harzianic acid and 2.0 × 10^−^^4^ M FeSO_4_ at different pH (from 3.96 to 8.46); (**B**) at different metal/harzianic acid molar ratio (from 0.00 to 1.49), at pH = 6.50 (**left** 2D–**right** 3D graphics).

**Figure 9:**
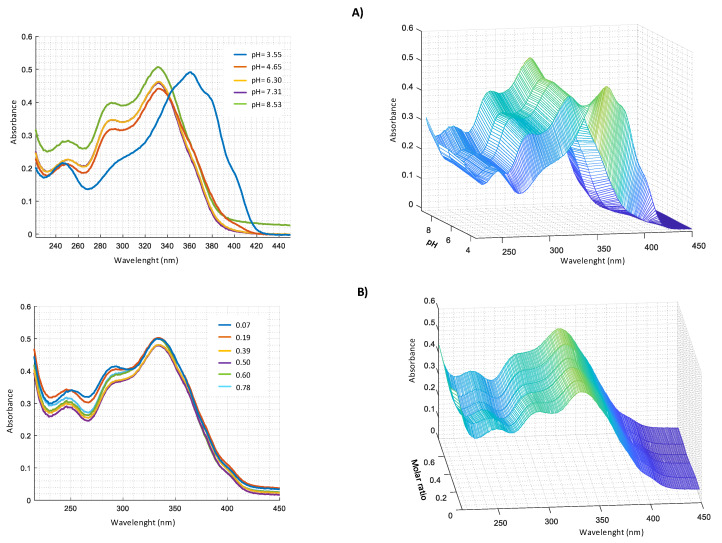
UV–VIS spectra in CH_3_OH/0.1 M NaClO_4_ (50:50 *w*/*w*) of: (**A**) 2.0 × 10^−^^4^ M harzianic acid and 2.0 × 10^−^^4^ M MnCl_2_ at different pH (3.55, 4.65, 6.30, 7.31, 8.53); (**B**) at different metal/harzianic acid molar ratio (0.70, 0.19, 0.39, 0.50, 0.60, 0.78) at pH = 6.50 (**left** 2D–**right** 3D graphics).

Similar measurements were performed in harzianic acid solutions with Cu^2+^, Fe^2+^, and Mn^2+^ (Figure 7, Figure 8 and Figure 9).

The collected data were processed numerically by Hyperquand program, obtaining the equilibrium constant reported in Table 2. The stability of stoichiometric complexes 1:2 follows the trend of the Irving–Williams series (Cu > Zn > Mn), while Fe^2+^ exhibits an anomalous behaviour due to the formation of different 1:2 complexes. A similar trend in the stability of the complexes also exists as a function of the first hydrolysis constant, as also observed by Martel and Hancock [31] for metal ions complexes with organic ligands (e.g., malonate and catechol).

**Figure 10:**
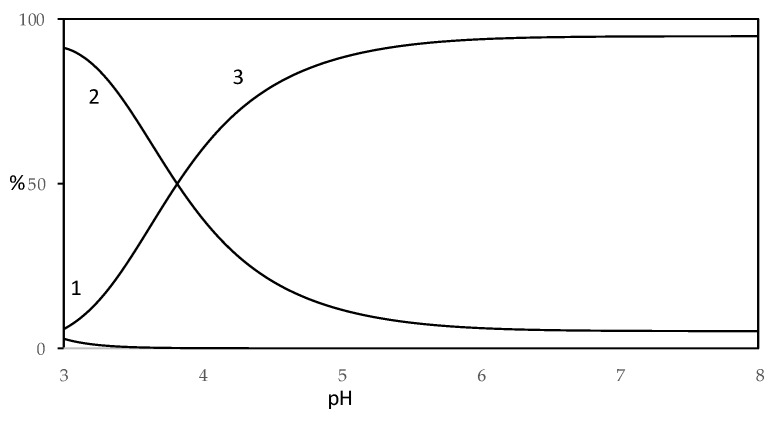
Distribution diagram of species for Cu(II)–HA system in CH_3_OH/0.1 M NaClO_4_ (50:50 *w*/*w* with 1.0 × 10^−4^ M Cu(II) and 2.0 × 10^−4^ M of HA acid (H_2_L) (1: Cu^2+^; 2: CuL; 3: CuL_2_^2^^−^).

**Figure 11:**
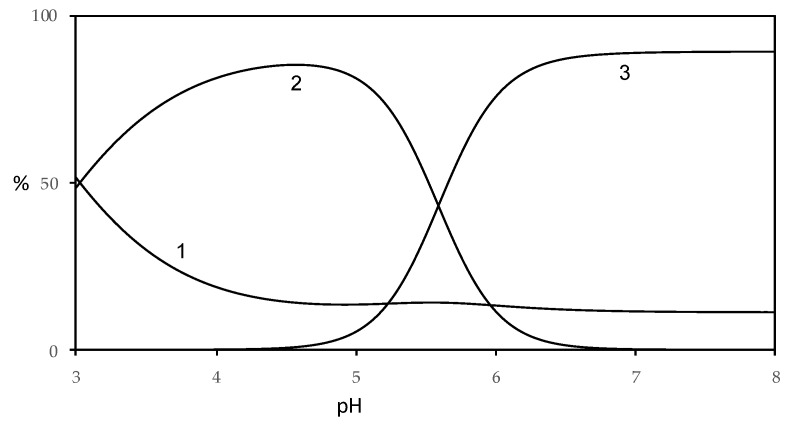
Distribution diagram of species for Fe(II)–harzianic acid system in CH_3_OH/0.1 M NaClO_4_ (50:50 *w*/*w* with 1.0 × 10^−4^ M Fe(II) and 2.0 × 10^−4^ M of harzianic acid (H_2_L) (1: Fe^2+^; 2: Fe(HL)_2_; 3: FeL_2_^2^^−^).

To visualize the amounts of the different species, distribution diagrams were built for Cu(II)–harzianic acid and Fe(II)– harzianic acid systems (Figure 10 and Figure 11).

From the distribution diagram of Cu(II)–harzianic acid system (metal ion/ligand ratio 1:2), reported in Figure 10, a prevalence of CuL is observed until pH 4, while for pH value higher than 4 there is a prevalence of CuL_2_^2^^+^ complex which reaches a maximum of about 90%. On the other hand, the CuL species is present in a non-significant amount (less than 10%).

By analysis of the distribution diagram of the Fe(II)–harzianic acid system (Figure 11; metal ion/ligand ratio 1:2), the free metal concentration decreases from 50%, at pH = 3, to about 10% for pH range 5–8 in favor of the formation of complex species. In particular, the Fe(HL)_2_ complex species becomes considerable for pH range 3–5.5 in amount until 85%. While the FeL_2_^2^^−^ complex species predominates in amount until 90% for pH higher than 5.5.

### 2.5. LC-MS and NMR Data

Chelating properties of harzianic acid were confirmed by LC-MS analysis of Metal (II)-HA. The main ions showed in MS spectra (Figures S7–S9) of each solution are reported in Table 3. In addition to ion characteristic of harzianic acid, LC-ESI-HRMS spectra of solutions in the presence of Cu(II), Mn(II) and Zn(II) exhibit dimeric peaks [2M − H + Metal]^+^ confirming the stoichiometric ratios observed in the previous section. Interestingly, the solution containing copper exhibits a peak at *m*/*z* 427.1057 corresponding to [M-H + Cu]^+^ and characteristic of a complex with stoichiometry 1:1. LC-MS analysis of harzianic acid with Fe(II) did not show any significant peak corresponding to complexes of this metal. Probably, this is due to the production of not charged species.

Finally, ^1^H-NMR spectra of harzianic acid solutions prepared with Mn^2+^ and Zn^2+^ confirmed the capacity of our compound to coordinate these cations. The analyses were recorded in CD_3_OD/D_2_O 1:1 (*w*/*w*), hence the proton spectrum of harzianic acid was previously acquired in the same solvent mixture for an accurate data interpretation. Chemical shifts were assigned on the basis of COSY experiment (Figures S10 and S11), and data reported in the literature [22]. In fact, ^1^H-NMR spectrum of harzianic acid (Figure S10) shows the loss of multiplicity of many signals and the overlap of H-8′ and H-6′B which resonates as multiplet at δ 2.12–1.96. In fact, the COSY spectrum showed a correlation between this signal and H-6′A multiplet at δ 2.41–2.32 and of H_3_-9′ and doublet H_3_-10′ resonating at δ 0.99. Moreover, the same spectrum showed a correlation between H_2_-7 (δ 1.53–1.51) and protons of the methyl group H_3_-8 (Table S1).

The ^1^H-NMR spectrum of harzianic acid recorded in presence of MnCl_2_ showed some significant shifts for H-3, H-4/5, H-5′ and H_3_-11′ resonating a δ 7.88 (dd, *J* =14.9, 11.3 Hz), 6.11-5.93 (m), 4.29 (d, *J* = 9.7 Hz) and 2.85 (s) respectively. In particular, downfield shift of H-3 and H-5′ of Δ δ 0.35 and 0.46, and upfield shift of H-4/5 and H_3_-11′ of Δδ 0.42 and 0.10, were respectively observed (Figure S12). A similar result is visible in the harzianic acid spectrum recorded in presence of Zn(ClO_4_)_2_ (Figure S12).

The coordination regioselectivity can be deduced by comparison between of NMR data of harzianic acid and its Cu(II) and Mn(II) complexes. In particular, the shifts of protons H-3 of the residue esadienoyl and H-5′ of the pyrrolidine ring could be related to the metal coordination with external carbonyl and amide groups.

## 3. Materials and Methods

### 3.1. Reagents and Their Analysis

Solutions of metals were prepared from Merck (Darmstadt, Germany) p.a. products [Cu(ClO_4_)_2_, Zn(ClO_4_)_2_, MnCl_2_ and FeSO_4_] dissolved in bidistilled water. The metal concentration was determined by complexometric method with a solution of EDTA at known concentration. Diluted solutions of perchloric acid were prepared from Merck p.a. products and standardized potentiometrically (glass electrode) against tris(hydroxymethyl)amino methane (Sigma-Aldrich, Saint Louis, MO, USA). The results agreed to within 0.1% or better.

Carbonate-free solutions of sodium hydroxide were obtained simply by diluting (after centrifugation) a saturated NaOH solution. To prevent air, contact the tubes were closed with a suba seal rubber. An approximately known quantity of NaOH was injected and immediately transferred under nitrogen atmosphere in a calibrated volumetric flask containing NaClO_4_ in the desired quantity, freshly boiled bidistilled water, and finally filled to the flask mark. The accurate hydroxide concentration was determined by titration with standard HClO_4_ using methyl red as a visual indicator. Replicated analyses agreed to within 0.1%.

6 molal solutions of sodium perchlorate (NaClO_4_·H_2_O, Merck p.a.). contain less than 10^−^^5^ molal concentration of iron, silica, heavy metals chloride, and sulphate ions. Stock solutions were analyzed gravimetrically by drying at 130 °C.

### 3.2. General Experimental Procedures

Optical rotations were measured in CH_3_OH using a Jasco P-1010 digital polarimeter (Jasco Corporation, Tokyo, Japan). NMR spectra were recorded at 400 MHz in CDCl_3_ or CD_3_OD or CD_3_OD/D_2_O 1:1 (*w*/*w*) on a Bruker spectrometer (Ascend^TM^400) (Bremen, Germany). The solvent was used as internal standard. The potentiometric titrations were performed in an air-bath thermostat kept at (25.00 ± 0.05) °C. A programmable computer-controlled data acquisition unit 3421A, supplied by Hewlett and Packard (Palo Alto, CA, USA), was used to perform the potentiometric measurements. The glass electrodes were Metrohm (Herisau, Switzerland) of 60102-100 type and Ag/AgCl electrode was utilized as reference. The EMF values were measured with a precision of ±0.01 mV using a Keithley 642 type Digital Electrometer (Tektronix Inc., Beaveron, OR, USA).

UV-VIS spectra were recorded by model Cary 5000 Spectrophotometer by Varian C. (Palo Alto, CA, USA), from 200 to 600 nm (optical path 0.2 cm) at 25.0 °C, under a constant flow of nitrogen. The far UV-CD spectra were recorded with a Jasco spectropolarimeter model J-715, from 200 to 600 nm (optical path 0.2 cm) at 25.0 °C, under a constant flow of nitrogen.

### 3.3. Isolation and Identification of Strain L1

Strain L1 was recovered from a specimen of the mollusc *Melarhaphe neritoides* (Gastropoda, Littorinidae) collected on an outcropping rock in the intertidal zone along the coastline of the isle of Procida, Bay of Naples, Italy. The small specimen was directly placed in a Petri dish containing potato dextrose agar (PDA: Hi Media, Mumbai, India) acidified with lactic acid (10 mL 10% l.a./L) and incubated at 25 °C in darkness. A hyphal tip from an emerging fungal colony was transferred in pure culture on PDA. The subculture developed very rapidly, covering the whole medium in 2 days; the surface promptly turned from white to yellow-green as sporulation progressed, indicative of belonging of L1 to the genus *Trichoderma*.

L1 was inoculated on PDA plates and cultivated for 7 days at 25 °C. Five mycelial plugs from actively growing cultures were used to inoculate 50 mL potato dextrose broth (PDB: Hi Media) in a 250 mL Erlenmeyer flask. Stationary culture was kept in the dark at 25 °C for 4 days. The biomass was collected by separating the substrate by filtration with Miracloth (Calbiochem, San Diego, CA, USA) paper, washed repeatedly with sterile distilled water, and dried with absorbent paper. The freeze-dried biomass was ground with a spatula and genomic DNA was isolated by using NucleoSpin^®^ Soil kit (Macherey-Nagel, Düren, Germany), following manufacturer’s instructions. DNA quantity was determined using a Qubit 2.0 fluorometer with the dsDNA BR Assay (Life Technologies, Grand Island, NY, USA). PCR analysis was carried out in 50 μL total reaction volume, with 0.5 μM primer, 0.2 mM dNTP Mix, 1× DreamTaq Green Buffer (Thermo Scientific, Waltham, MA, USA), and 1.25 U of DreamTaq DNA Polymerase (Thermo Scientific). The translation elongation factor -1 alpha (TEF-1α) was amplified by the primer pair TEF-1f (ATGGGTAAGGARGACAAGAC) and TEF-1r (GGARGTACCAGTSATCATGTT). The products obtained were analyzed by subjecting 5 µL of them to gel electrophoresis of agarose. The PCR product was purified with the PureLink Quick PCR Purification (Invitrogen, Waltham, MA, USA) kit, following the manufacturer′s instructions. Sequencing reactions were performed by Eurofins Genomics (Eurofins Genomics analysis laboratories, Vimodrone, Milano, Italy) with the same primer sets used for PCR amplification. The nucleotide sequence of the inserts thus obtained was subjected to in silico analysis through the BlastX program which compares the nucleotide sequence with those contained in the GenBank database (Genbank National Center for Biotechnology Information U.S. National Library of Medicine Rockville Pike, Bethesda, MD, USA) of the National Center for Biotechnological information. Sequence has been deposited in GenBank under the reference numbers MT309187.

### 3.4. Production of Culture Filtrates and HA extraction

Mycelial plugs from actively growing cultures on PDA of strain L1 were used to inoculated in 1 L-Erlenmayer flasks containing 500 mL PDB. Cultures were kept in darkness at 25 °C for 3 weeks, then filtered through filter paper (Whatman, Maidstone, UK). The culture filtrate (1 L) was acidified to pH 2 with 2 N HCl and extracted three time with the same volume of ethyl acetate (EtOAc). The organic extracts were combined, dried with Na_2_SO_4_, and evaporated under reduced pressure to give a brown-red oil residue. The extract was dissolved in CHCl_3_ and then extracted with a saturated solution of NaHCO_3_. The aqueous phase was acidified at pH 2 with concentrated HCl and extracted three times with EtOAc. The organic phase obtained was dried with Na_2_SO_4_, and evaporated under reduced pressure affording a red-brown solid residue (150.7 mg) (Figure 2).

### 3.5. LC-MS Analysis

In order to investigate binding properties of harzianic acid towards bivalent metal, 500μL of each metal water solution (2 mM) were added to 500 μL of a methanolic solution of harzianic acid (1 mg/mL) and directly infused into the LC-MS system. Analyses were done on an Agilent high performance liquid chromatograph (HPLC) 1260 Infinity Series (Agilent Technologies, Santa Clara, CA, USA) coupled to a quadrupole-time of flight (Q-TOF) mass spectrometer model G6540B (Agilent Technologies) with a Dual ESI source (Agilent Technologies). Samples were injected in 7 μL injection volumes and eluted at 100% of 0.1% formic acid in acetonitrile at a flow rate of 0.3 mL/min to the mass spectrometer. MS parameters were set using the Agilent MassHunter Data Acquisition Software, rev. B.05.01 (Agilent Technologies). The system operated in positive ion mode and MS spectra were recorded in *m*/*z* 50–1000 range as centroid spectra, with a speed of 3.3 spectra/s. The capillary was maintained at 2000 V, fragmentor voltage at 180 V, cone 1 (skimmer 1) at 45 V, Oct RFV at 750 V. Gas flow rate was set at 11 L/min, at 350 °C, and the nebulizer was set at 45 psig. A standard solution was infused by using an Isocratic pump (1260 Infinity Series, Agilent Technologies) in order to perform the real-time lock mass correction. The solution consisted of two reference mass compounds: purine (C_5_H_4_N_4_ at *m*/*z* 121.050873, 10 µmol/L) and hexakis (1H,1H, 3H-tetrafluoropentoxy)-phosphazene (C_18_H_18_O_6_N_3_P_3_F_24_ at *m*/*z* 922.009798, 2 µmol/L). Flow rate was set at 0.06 mL/min, while the detection window and the minimum height were set at 1000 ppm and 10,000 counts, respectively, for reference mass correction.

Analyses of L1 culture filtrates were done on the Agilent system previously reported. Separations were performed on a Zorbax Eclips Plus C18 column, 4.6 × 100 mm, with 3.5 µm particles (Agilent Technologies). Analyses were done at a constant temperature of 37 °C and using a linear gradient system composed of A: 0.1% (*v*/*v*) formic acid in water, and B: 0.1% (*v*/*v*) formic acid in acetonitrile. The flow was 0.6 mL min^−1^, 95% A graduating to 100% B in 6 min, 100% B 6–8 min, 95% A 8–10.

### 3.6. Potentiometric and Spectrophotometric Measurements

The solutions tested (TS_H_) had the following composition TS_H_: C_L_ M H_2_L, C_H_ M HClO_4_, C_OH_ M NaOH, (0.1–C_H_) M NaClO_4_/CH_3_OH 50:50 (*w*/*w*). The hydrogenionic concentration [H^+^] was measured using the following cell:

$$RE\;/\;TS_{H}\;/\;GE$$

where GE represents the glass electrode, while RE is the composition reference cell: Ag _(S)_/AgCl _(S)_/0.1 M NaClO_4_/CH_3_OH (50:50). The e.m.f. of the cell at 25 °C, results:

(1) $$E_{G}={E_{G}^{0}}^{\prime }+0.05916\cdot log\left(h\cdot yH\right)$$

where [H^+^] = *h*, ${E_{G}^{0}}^{\prime }$ is a constant in each titration, *y_H_* represents the activity coefficient of H^+^ which is constant in the ionic medium used. *E_J_* is the liquid junction potential that is generated on contact between the solution 0.1 M NaClO_4_ / CH_3_OH (50:50) and the measuring solution [32]. Putting:

(2) $$E_{G}={E_{G}^{0}}^{\prime }+0.05916\cdot log\left(yH\right)+E_{J}$$

Equation (1) takes the form:

(3) $$E_{G}=E_{G}^{0}+0.05916\cdot log\left(h\right)$$

The experimental measurements were carried out in the form of both potentiometric and spectrophotometric titration. Each titration was divided into two parts: in the first, the constant $E_{G}^{0}$ was determined in a solution in the absence of harzianic acid and metal ion. In the second part, the harzianic acid and metal ion were added and the acidity of the solution was decreased by addition of a NaOH solution. After each addition, the e.m.f. of cell is measured determining the hydrogenionic concentration h (and therefore the pH) and at the same time, the UV-VIS spectrum of the resulting solution is recorded. Hyperquad program (Protonic Software, Leeds, UK) [33] was used to process the experimental data obtained by potentiometric and spectrophotometric measurements.

## 4. Conclusions

In this paper production of harzianic acid by *T. pleuroticola* is reported for the first time. Considering the recent separation of this specie from *T. harzianum*, it would be interesting to evaluate if the ability to synthesize this tetramic acid occurs in other strains, and whether or not harzianic acid can be regarded as a chemotaxonomic marker of any species within the *T. harzianum* aggregate.

The bioactivities of acetylated tetramic acids are linked to their capacity to form complexes with ions and to their acidic properties. In general, studies conducted on these complexes, such as those concerning tenuazoic acid and cyclopiazonic acid [24] underrate the relevance of the formation conditions and of complex stabilities. Acylated tetramic acids are synthesized by hybrid polyketide synthase-nonribosomal peptide synthetase enzymes (PKS-NRPS; NRPS-PKS) and the properties of this class of compounds may explain their antibiotic activity [24,34]. Metal-chelating metabolites can inhibit virus-induced or microbial-induced enzymes in infected cells by coordinating with metals at their active sites with a significant pharmaceutical impact [24,35]. Moreover, agricultural applications have also been demonstrated in the capability of harzianic acid to cause Fe(III)-promoting plant growth due to the iron solubilization [21,23].

In the present work, physico-chemical properties of harzianic acid were investigated, such as pKa, chelating properties toward metals with relevant biological functions, and stability constants of the derived complexes. Our findings show the coordination capacity of harzianic acid toward Cu(II), Fe(II), Mn(II) and Zn(II) through the formation of neutral or charged complexes in metal(II)/harzianic acid ratio of 1:1 or 1:2 depending on the pH conditions, as observed for Cu(II) and Fe(II), or in 1:2 ratio as observed for Mn(II) and Zn(II). In particular, the highest affinity constant is obtained for the coordination complex Cu(II)/harzianic acid 1:2 with log β 15.82.

## Figures and Tables

**Table 1:** Summary of acidity constants (cologarithms) of harzianic acid (H_2_L), obtained by potentiometric and spectrophotometric measurements.

Equilibria	Dissociation of Acid Group	Spectrophotometry	Potentiometry
H_2_L + H_2_O = HL^−^ + H_3_O^+^	R-COO^−^	4.08 ± 0.02	4.00 ± 0.09
HL^−^ + H_2_O = L^2−^ + H_3_O^+^	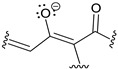	5.63 ± 0.08	5.9 ± 0.2

**Table 2:** Summary of metal ion/harzianic acid (H_2_L) stability constants.

Me^2+^	Equilibria	log (cost.eq.) ± 3σ *
Cu^2+^	Cu^2+^ + L^2^^−^ = CuL	9.26 ± 0.08
	Cu^2+^ + 2 L^2^^−^ = CuL_2_^2^^+^	15.82 ± 0.05
Zn^2+^	Zn^2+^ + 2 L^2^^−^ = ZnL_2_^2^^+^	14.42 ± 0.06
Mn^2+^	Mn^2+^ + 2 L^2^^−^ = MnL_2_^2^^+^	11.96 ± 0.09
Fe^2+^	Fe^2+^ + 2 HL^−^ = Fe(HL)_2_	13.20 ± 0.04
	Fe^2+^ + 2 L^2^^−^ = FeL_2_^2^^+^	10.22 ± 0.07

* denote the estimated standard deviation.

**Table 3:** Caracterization of Metal^2+^-harzianic acid complex by LC/MS.

Ion	Experimental Mass	Formula	Exact Mass
**Harzianic acid: Cu(ClO_4_)_2_**			
[M + H]^+^	366.1921	C_19_H_28_NO_6_	366.1917
[M + Na]^+^	388.1730	C_19_H_27_NO_6_Na	388.1736
[M – H + Cu]^+^	427.1057	C_19_H_26_NO_6_Cu	427.1056
[M + Cu + ClO_4_]^+^	527.0619	C_19_H_27_NO_10_CuCl	527.0619
[2M – H + Cu]^+^	792.2880	C_38_H_53_N_2_O_12_Cu	792.2895
**Harzianic Acid: MnCl_2_**			
[M + H]^+^	366.1908	C_19_H_28_NO_6_	366.1917
[M + Na]^+^	388.1728	C_19_H_27_NO_6_Na	388.1736
[2M + Na]^+^	753.3549	C_38_H_54_N_2_O_12_Na	753.3574
[2M – H + Mn]^+^	784.2952	C_38_H_53_N_2_O_12_Mn	784.2979
**Harzianic Acid: Zn(ClO_4_)_2_**			
[M + H]^+^	366.1922	C_19_H_28_NO_6_	366.1917
[M + Na]^+^	388.1738	C_19_H_27_NO_6_Na	388.1736
[M + Zn + ClO_4_]^+^	528.0612	C_19_H_27_NO_10_ZnCl	528.0615
[2M – H + Zn]^+^	793.2886	C_38_H_53_N_2_O_12_Zn	793.2890

## Supplementary Materials

The following are available online, Figure S1: ITS sequence of Trichoderma pleuroticola L1, Table S1: NMR data of harzianic acid recorded in different solvents, Figure S2: LC-MS mass spectrum of harzianic acid, Figure S3: NMR spectrum of harzianic acid recorded in CDCl3, Figures S4 and S5: NMR spectra of harzianic acid recorded in CD3OD, Figure S6: UV-VIS spectra for solutions of harzianic acid, Figures S7–S9: mass spectra ESIMS QTOF of harzianic acid in presence of metals, Figures S10 and S11: NMR spectra of harzianic acid record in CD3OD/D2O, Figure S12: NMR spectra of harzianic acid in presence of metals.

## References

[1] Harman G.E., Howell C.R., Viterbo A., Chet I., Lorito M. (2004). *Trichoderma* species—Opportunistic, avirulent plant symbionts. Nat. Rev. Microbiol..

[2] Vinale F., Sivasithamparam K., Ghisalberti E.L., Marra R., Woo S.L., Lorito M. (2008). *Trichoderma*–plant–pathogen interactions. Soil Biol. Biochem..

[3] Kredics L., Hatvani L., Naeimi S., Körmöczi P., Manczinger L., Vágvölgyi C., Druzhinina I., Gupta V.K., Schmoll M., Herrera-Estrella A., Upadhyay R.S., Druzhinina I., Tuohy M.G. (2014). Biodiversity of the genus *Hypocrea**/Trichoderma* in different habitats. Biotechnology and Biology of Trichoderma.

[4] Nicoletti R., Vinale F. (2018). Bioactive compounds from marine-derived *Aspergillus*, *Penicillium*, *Talaromyces* and *Trichoderma* Species. Mar. Drugs.

[5] Reino J.L., Guerrero R.F., Hernández-Galán R., Collado I.G. (2008). Secondary metabolites from species of the biocontrol agent *Trichoderma*. Phytochem. Rev..

[6] Zeilinger S., Gruber S., Bansal R., Mukherjee P.K. (2016). Secondary metabolism in *Trichoderma*–Chemistry meets genomics. Fungal Biol. Rev..

[7] Marra R., Nicoletti R., Pagano E., DellaGreca M., Salvatore M.M., Borrelli F., Lombardi N., Vinale F., Woo S.L., Andolfi A. (2018). Inhibitory effect of trichodermanone C, a sorbicillinoid produced by *Trichoderma citrinoviride* associated to the green alga *Cladophora* sp., on nitrite production in LPS-stimulated macrophages. Nat. Prod. Res..

[8] Song Y.P., Miao F.P., Fang S.T., Yin X.L., Ji N.Y. (2018). Halogenated and nonhalogenated metabolites from the marine-alga-endophytic fungus *Trichoderma asperellum* cf44-2. Mar. Drugs.

[9] Meng J., Cheng W., Heydari H., Wang B., Zhu K., Konuklugil B., Lin W. (2018). Sorbicillinoid-based metabolites from a sponge-derived fungus *Trichoderma saturnisporum*. Mar. Drugs.

[10] Yamada T., Fujii A., Kikuchi T. (2019). New diterpenes with a fused 6-5-6-6 ring system isolated from the marine sponge-derived fungus *Trichoderma harzianum*. Mar. Drugs.

[11] Salvatore M.M., Nicoletti R., DellaGreca M., Andolfi A. (2019). Occurrence and properties of thiosilvatins. Mar. Drugs.

[12] Marfori E.C., Kajiyama S., Fukusaki E., Kobayashi A. (2002). Trichosetin, a novel tetramic acid antibiotic produced in dual culture of *Trichoderma harzianum* and *Catharanthus roseus* callus. Z. für Naturforschung C J. Biosci..

[13] Mo X., Li Q., Ju J. (2014). Naturally occurring tetramic acid products: Isolation, structure elucidation and biological activity. RSC Adv..

[14] Sawa R., Mori Y., Iinuma H., Naganawa H., Hamada M., Yoshida S., Furutani H., Kajimura Y., Fuwa T., Takeuchi T. (1994). Harzianic acid, a new antimicrobial antibiotic from a fungus. J. Antibiot..

[15] Kang D., Kim J., Choi J.N., Liu K.H., Lee C.H. (2011). Chemotaxonomy of *Trichoderma* spp. using mass spectrometry-based metabolite profiling. J. Microbiol. Biotechnol..

[16] Vinale F., Manganiello G., Nigro M., Mazzei P., Piccolo A., Pascale A., Ruocco M., Marra R., Lombardi N., Lanzuise S. (2014). A novel fungal metabolite with beneficial properties for agricultural applications. Molecules.

[17] Kawada M., Yoshimoto Y., Kumagai H., Someno T., Momose I., Kawamura N., Isshiki K., Ikeda D. (2004). PP2A inhibitors, harzianic acid and related compounds produced by fungus strain F-1531. J. Antibiot..

[18] Vinale F., Nicoletti R., Borrelli F., Mangoni A., Parisi O.A., Marra R., Lombardi N., Lacatena F., Grauso L., Finizio S. (2017). Co-culture of plant beneficial microbes as source of bioactive metabolites. Sci. Rep..

[19] Chaverri P., Branco-Rocha F., Jaklitsch W., Gazis R., Degenkolb T., Samuels G.J. (2015). Systematics of the *Trichoderma harzianum* species complex and the re-identification of commercial biocontrol strains. Mycologia.

[20] Kubicek C.P., Steindorff A.S., Chenthamara K., Manganiello G., Henrissat B., Zhang J., Cai F., Kopchinkiy A.G., Kubicek E.M., Kuo A. (2019). Evolution and comparative genomics of the most common *Trichoderma* species. BMC Genom..

[21] Vinale F., Flematti G., Sivasithamparam K., Lorito M., Marra R., Skelton B.W., Ghisalberti E.L. (2009). Harzianic acid, an antifungal and plant growth promoting metabolite from *Trichoderma harzianum*. J. Nat. Prod..

[22] Healy A.R., Vinale F., Lorito M., Westwood N.J. (2015). Total synthesis and biological evaluation of the tetramic acid based natural product harzianic acid and its stereoisomers. Org. Lett..

[23] Vinale F., Nigro M., Sivasithamparam K., Flematti G., Ghisalberti E.L., Ruocco M., Varlese R., Marra R., Lanzuise S., Eid A. (2013). Harzianic acid: A novel siderophore from *Trichoderma harzianum*. FEMS Microbiol. Let..

[24] Zaghouani M., Nay B. (2016). 3-Acylated tetramic and tetronic acids as natural metal binders: Myth or reality?. Nat. Prod. Rep..

[25] Hood M.I., Skaar E.P. (2012). Nutritional immunity: Transition metals at the pathogen–host interface. Nat. Rev. Microbiol..

[26] Błaszczyk L., Siwulski M., Sobieralski K., Frużyńska-Jóźwiak D. (2013). Diversity of *Trichoderma* spp. causing *Pleurotus* green mould diseases in Central Europe. Folia Microbiol..

[27] Park M.S., Bae K.S., Yu S.H. (2006). Two new species of *Trichoderma* associated with green mold of oyster mushroom cultivation in Korea. Mycobiology.

[28] Korkmaz M.N., Ozdemir S.C., Uzel A. (2017). Xylanase production from marine derived *Trichoderma pleuroticola* 08ÇK001 strain isolated from Mediterranean coastal sediments. J. Basic Microbiol..

[29] McConnell H. (1952). Effect of polar solvents on the absorption frequency of n→ π electronic transitions. J. Chem. Phys..

[30] Banerjee S., Bhanja S.K., Chattopadhyay P.K. (2018). Quantum chemical predictions of aqueous pKa values for OH groups of some α-hydroxycarboxylic acids based on ab initio and DFT calculations. Comput. Theor. Chem..

[31] Martell A.E., Hancock R.D. (1996). Metal Complexes in Aqueous Solutions, Modern Inorganic Chemistry.

[32] Rochester C.H. (1972). The ionic products of water and methanol in methanol–water mixtures. J. Chem. Soc. Dalton Trans..

[33] Gans P., Sabatini A., Vacca A. (1996). Investigation of equilibria in solution. Determination of equilibrium constants with the HYPERQUAD suite of programs. Talanta.

[34] Ghisalberti E.L., Atta-ur R. (2003). Bioactive tetramic acid metabolites. Studies in Natural Products Chemistry.

[35] Hutchinson D.W. (1985). Metal chelators as potential antiviral agents. Antivir. Res..

