# Supplementary material for: Bivalent Metal-Chelating Properties of Harzianic Acid Produced by Trichoderma pleuroticola Associated to the Gastropod Melarhaphe neritoides
Source: Molecules. 2020 May 4;25(9):2147. doi: 10.3390/molecules25092147 (PMC7248884; doi:10.3390/molecules25092147)
Supplement: Supplementary file 1 [file molecules-25-02147-s001.pdf]

Bivalent Metal-Chelating Properties of Harzianic Acid Produced by *Trichoderma pleuroticola* Associated to the Gastropod *Melarhaphe neritoides*

Gaetano De Tommaso [1], Maria Michela Salvatore [1], Rosario Nicoletti [2,3], Marina DellaGreca [1], Francesco Vinale [4,5], Assunta Bottiglieri [3], Alessia Staropoli [3,5], Francesco Salvatore [1], Matteo Lorito [3,5], Mauro Iuliano [1,*] and Anna Andolfi [1,*]

1 Department of Chemical Sciences, University of Naples Federico II, 80126 Naples, Italy; gaetano.detommaso@unina.it (G.D.T.); mariamichela.salvatore@unina.it (M.M.S.); dellagre@unina.it (M.D.); frsalvat@unina.it (F.S.)
2 Council for Agricultural Research and Economics, Research Centre for Olive, Fruit and Citrus Crops, 81100 Caserta, Italy; rosario.nicoletti@crea.gov.it
3 Department of Agricultural Sciences, University of Naples Federico II, 80055 Portici, Italy; assunta_bottiglieri@libero.it (A.B.); alessia.staropoli@unina.it (A.S.); matteo.lorito@unina.it (M.L.)
4 Department of Veterinary Medicine and Animal Productions, University of Naples Federico II, 80137 Naples, Italy; frvinale@unina.it
5 Institute for Sustainable Plant Protection, National Research Council, 80055 Portici, Italy
* Correspondence: mauro.iuliano@unina.it (M.I.); andolfi@unina.it (A.A.); Tel.: +39-081-2539179 (A.A.)

Figure S1. ITS sequence of *Trichoderma pleuroticola* L1.
Table S1. ^{1}H NMR data of harzianic acid recorded in different solvents.
Figure S2. Harzianic acid mass spectrum obtained by LC-MS analysis in ESI positive mode.
Figure S3. ^{1}H NMR spectrum of harzianic acid recorded in $CDCl_3$ at 400 MHz
Figure S4. ^{1}H NMR spectrum of harzianic acid recorded in CD_3OD at 400 MHz
Figure S5. COSY spectrum of harzianic acid recorded in CD_3OD at 400 MHz
Figure S6. UV–Vis spectra for solutions: $5.5 \cdot 10^{-4}$ M harzianic acid in CH_3OH; $2.6 \cdot 10^{-4}$ M harzianic acid in 2–propanol (optical path 0.1 cm).
Figure S7. Mass spectrum ESIMS QTOF obtained by LC-MS analysis in ESI positive mode of a solution $1.0 \cdot 10^{-3}$ M of harzianic acid and $1.0 \cdot 10^{-3}$ M of $Cu(ClO_4)_2$ in MeOH/H_2O 50:50 (*w*/*w*).
Figure S8. Mass spectrum ESIMS QTOF obtained by LC-MS analysis in ESI positive mode of a solution $1.0 \cdot 10^{-3}$ M of harzianic acid and $1.0 \cdot 10^{-3}$ M of $MnCl_2$ in MeOH/H_2O 50:50 (*w*/*w*).
Figure S9. Mass spectrum ESIMS QTOF obtained by LC-MS analysis in ESI positive mode of a solution $1.0 \cdot 10^{-3}$ M of harzianic acid and $1.0 \cdot 10^{-3}$ M of $Zn(ClO_4)_2$ in MeOH/H_2O 50:50 (*w*/*w*).
Figure S10. ^{1}H NMR spectrum of harzianic acid recorded in CD_3OD/D_2O (50:50 *w*/*w*) at 400 MHz
Figure S11. COSY spectrum of harzianic acid recorded in CD_3OD/D_2O (50:50 *w*/*w*) at 400 MHz
Figure S12. Detail of ^{1}H NMR spectra of harzianic acid (down), Zn^{2+}-HA (middle) and Mn^{2+}-HA (up) recorded in CD_3OD/D_2O (50:50 *w*/*w*) at 400 MHz. Solvent peak is removed.

TCTTTTCCCTCGCGTCGGCATCATTCGCCGCTCTGATTCTCCAGACACTTGTGCTAACCG
TCTCCTAGGGGTGCGTATTCCATGAATCCCCTTGAATGTGATCGATCGAACACAACACTG
ACTTGTTACAACAGCCACGTCGACTCCGGCAAGTCGACCACCGTGAGTTGCACCCCCTTC
TCTTGCTCCGGCATCAAGCGAGAGCGTTTGATGCCGGACATGTACTCTTTTGAACCCAGG
GCTGACCATTCATCATGTAGACTGGTCACTTGATCTACCAGTGCGGTGGTATCGATCGTC
GTACCATCGAGAAGTTCGAGAAGGTAAGCTTCAACTGATTTTCGCCTCAATTCTCCCTCC
ACATTCAATTGTGCCCGACGATTCTGCAGAGAATTTTCGTGTCGACAATTTTTCATCACC
CCGCTTTCCGTTACCCCTCCTTTGGCAGCGACGCAAATTTTTTGTGGCAGCCTTTTGGCT
TTGGTGGGGTTTCGTGTGCACCCCACTAGCTCACTGCTTTTTTCTGCTTCACTCTCCCAC
TGCCCAGTCATCATTCAACGTGCTCTGTGTCTCGCCATTCAGCGATGCTAACCACTTTTC
CATCAATAGGAAGCCGCCGAACTCGGCAAGGGTTCCTTCAAGTACGCTTGGGTTCTTGAC
AAGCTCAAGGCCGAGCGTGAGCGTGGTATCACCATCGACATTGCCCTGTGGAAGTTCGAG
ACTCCCAAGTACTATGTCACCGTCATTGGTATGTCTGCTCCATCATCTTGATGCAGGAAT
TGCGAGCTGGTGTTAACAGGTAATTCGCAGATGCTCCCGGCCACCGTGATTTCATCA

Figure S1. ITS sequence of *Trichoderma pleuroticola* L1.

Table 1. ^{1}H NMR data of harzianic acid recorded in different solvents.

Position	δ_H (*J* in Hz) $CDCl_3$[a]	δ_H (*J* in Hz) CD_3OD[1]	δ_H (*J* in Hz) CD_3OD/D_2O
2	7.02 d (15.2)	7.12 d (15.2)	7.14-6.99 brs
3	7.60-7.55 m	7.58-7.52 brs	7.68-7.46 brs
4	6.41-6.38 m[b]	6.44-6.38 m[b]	6.58-6.32 brs[b]
5	6.38-6.36 m[b]	6.44-6.38 m[b]	6.58-6.32 brs[b]
6	2.29-2.21 m	2.30-2.20 m	2.30-2.17 brs
7	1.51 sest (7.4)	1.52 sept (7.4)	1.53-1.51 m
8	0.97 t (7.3)	1.01-0.95 m[b]	0.99 m[b]
5′	3.65 brd (10.2)	3.83 dd (8.6, 2.0)	3.91-3.82 brs
6′A	2.50 brd (14.3)	2.36 dd 14.6, 2.0	2.41-2.32 brs
6′B	1.93 dd (14.3, 10.2)	2.00 m[b]	2.12-1.96 br[b]
8′	2.03 sept (6.8)	2.05 m[b]	2.12-1.96 brs[b]
9′	1.00 d (6.8)	2.96 s	2.96 s
10′	1.00 d (6.8)	1.01-0.95 m[2]	0.99 m[2]
11′	3.00 s		

[a]Spectroscopic data are in accordance with those previously reported (Healy et al., 2015)

[b]Overlapping signals

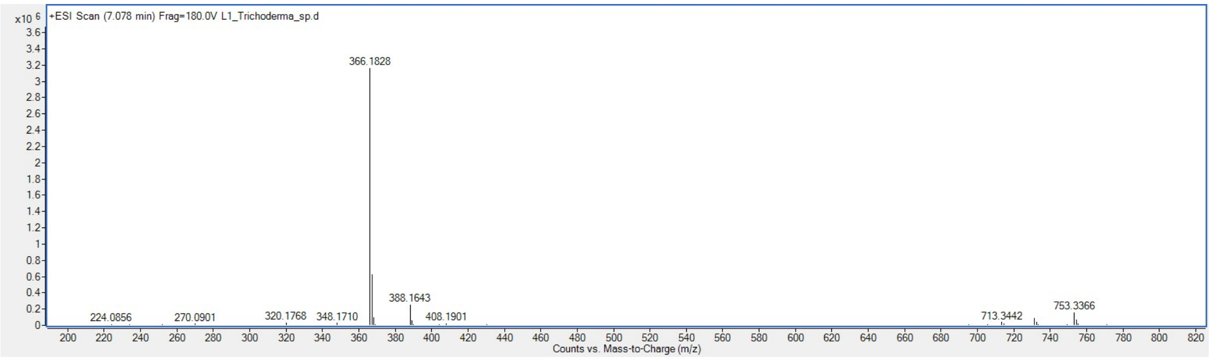

Figure S2. Harzianic acid mass spectrum obtained by LC-MS analysis in ESI positive mode.

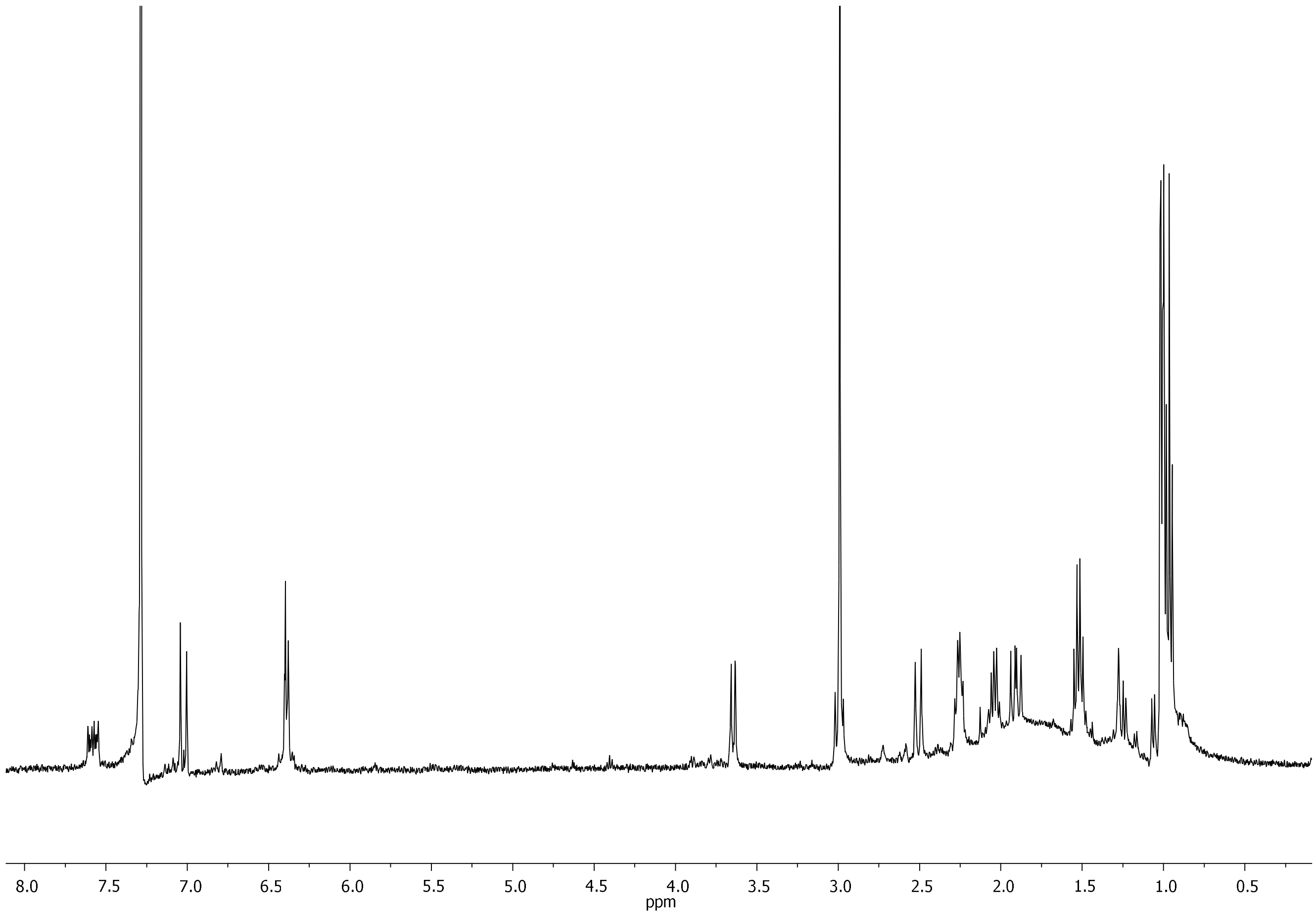

Figure S3. ^{1}H NMR spectrum of harzianic acid recorded in $CDCl_3$ at 400 MHz

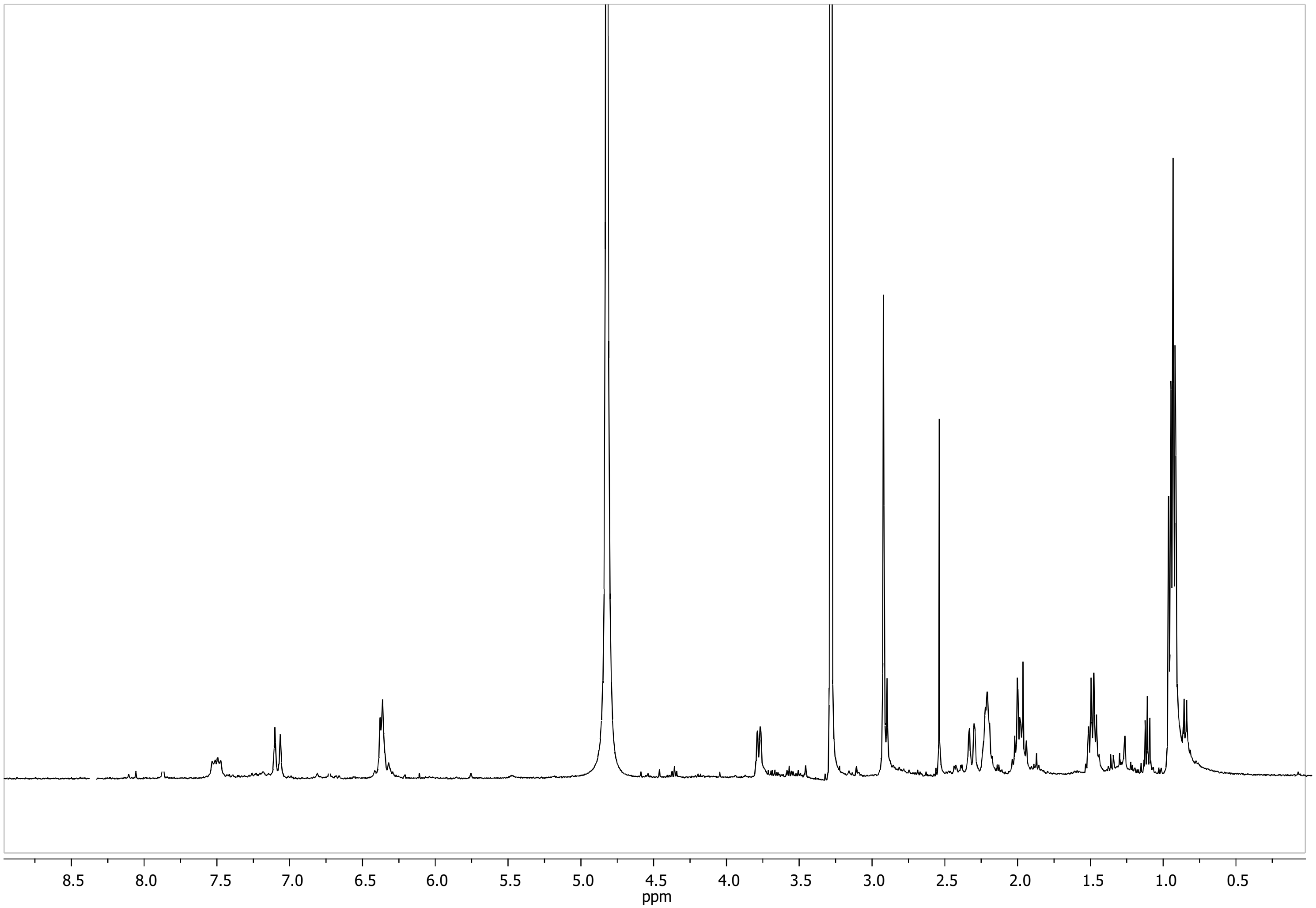

Figure S4. ^{1}H NMR spectrum of harzianic acid recorded in CD_3OD at 400 MHz

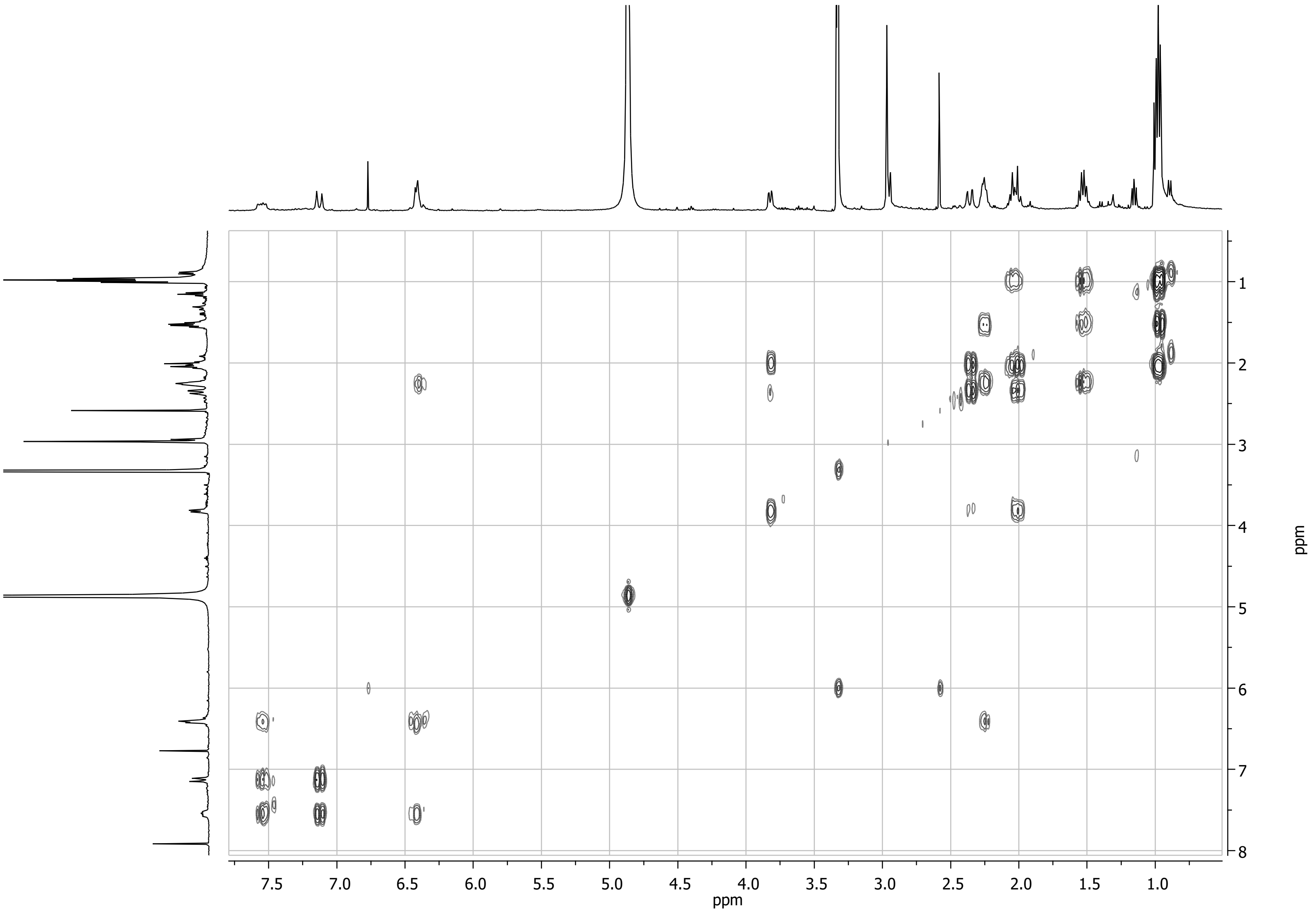

Figure S5. COSY spectrum of harzianic acid recorded in CD_3OD at 400 MHz

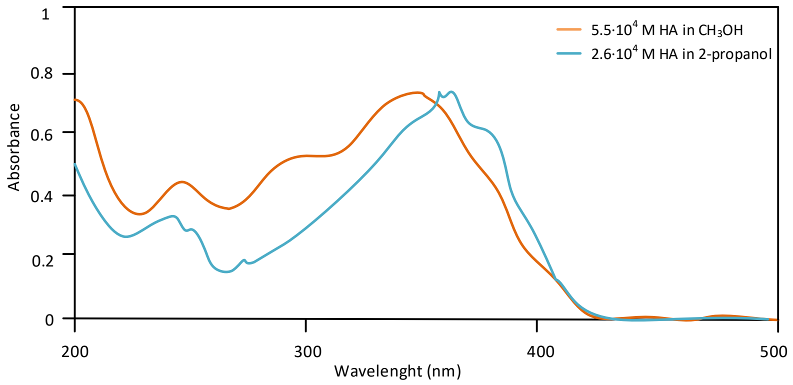

Figure S6. UV–Vis spectra for solutions: 5.5·10^{-4} M harzianic acid in CH_3OH; 2.6·10^{-4} M harzianic acid in 2–propanol (optical path 0.1 cm).

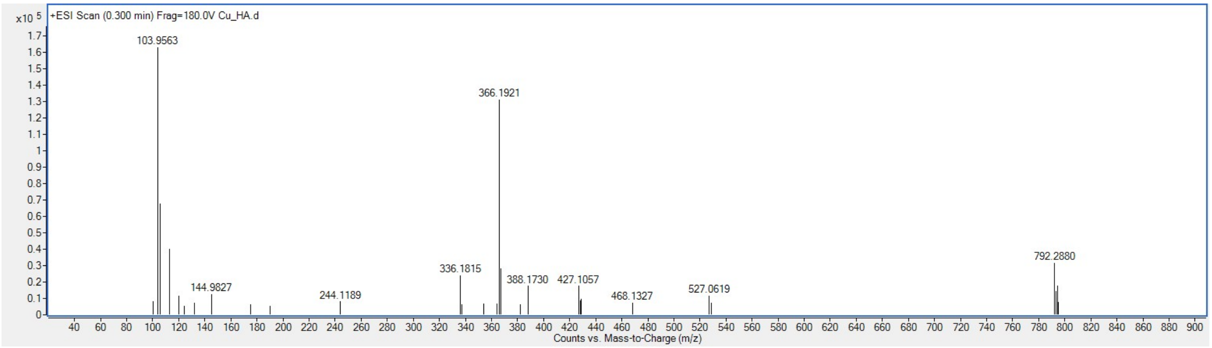

Figure S7. Mass spectrum ESIMS QTOF obtained by LC-MS analysis in ESI positive mode of a solution $1.0 \cdot 10^{-3}$ M of harzianic acid and $1.0 \cdot 10^{-3}$ M of $Cu(ClO_4)_2$ in MeOH/H_2O 50:50 (*w*/*w*).

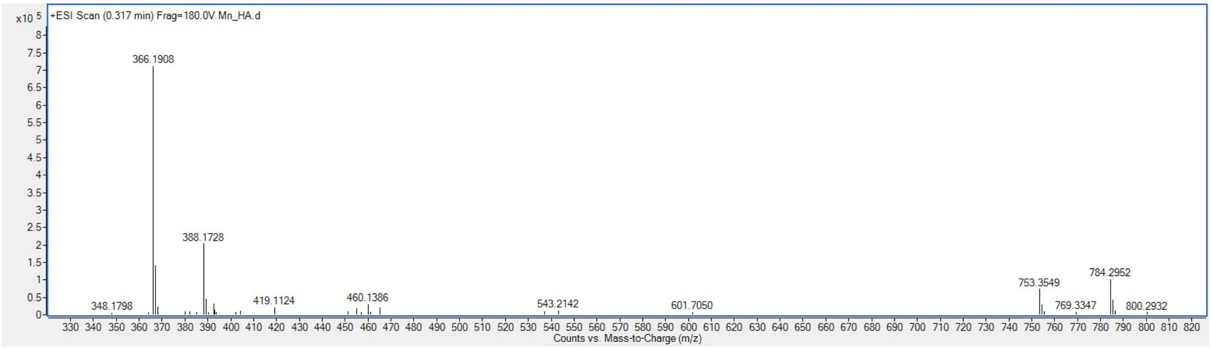

Figure S8. Mass spectrum ESIMS QTOF obtained by LC-MS analysis in ESI positive mode of a solution $1.0 \cdot 10^{-3}$ M of harzianic acid and $1.0 \cdot 10^{-3}$ M of $MnCl_2$ in MeOH/H_2O 50:50 (*w*/*w*).

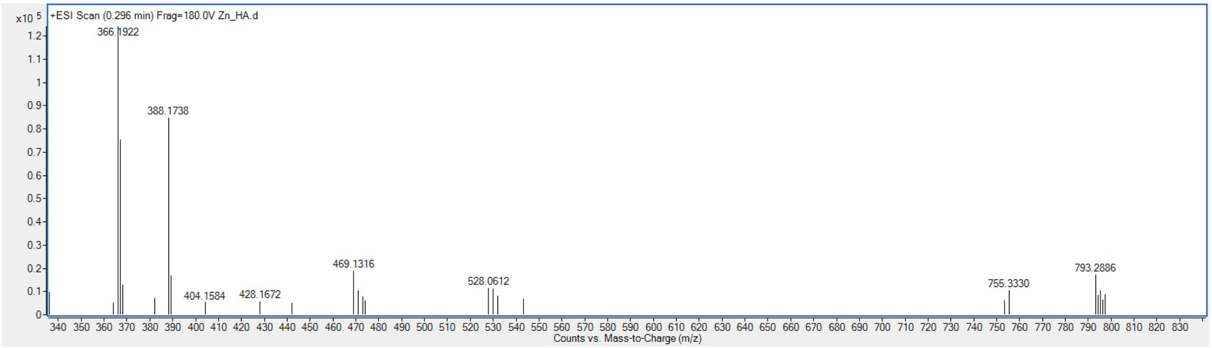

Figure S9. Mass spectrum ESIMS QTOF obtained by LC-MS analysis in ESI positive mode of a solution $1.0{\cdot}10^{-3}$ M of harzianic acid and $1.0{\cdot}10^{-3}$ M of $Zn(ClO_4)_2$ in MeOH/H_2O 50:50 (*w*/*w*).

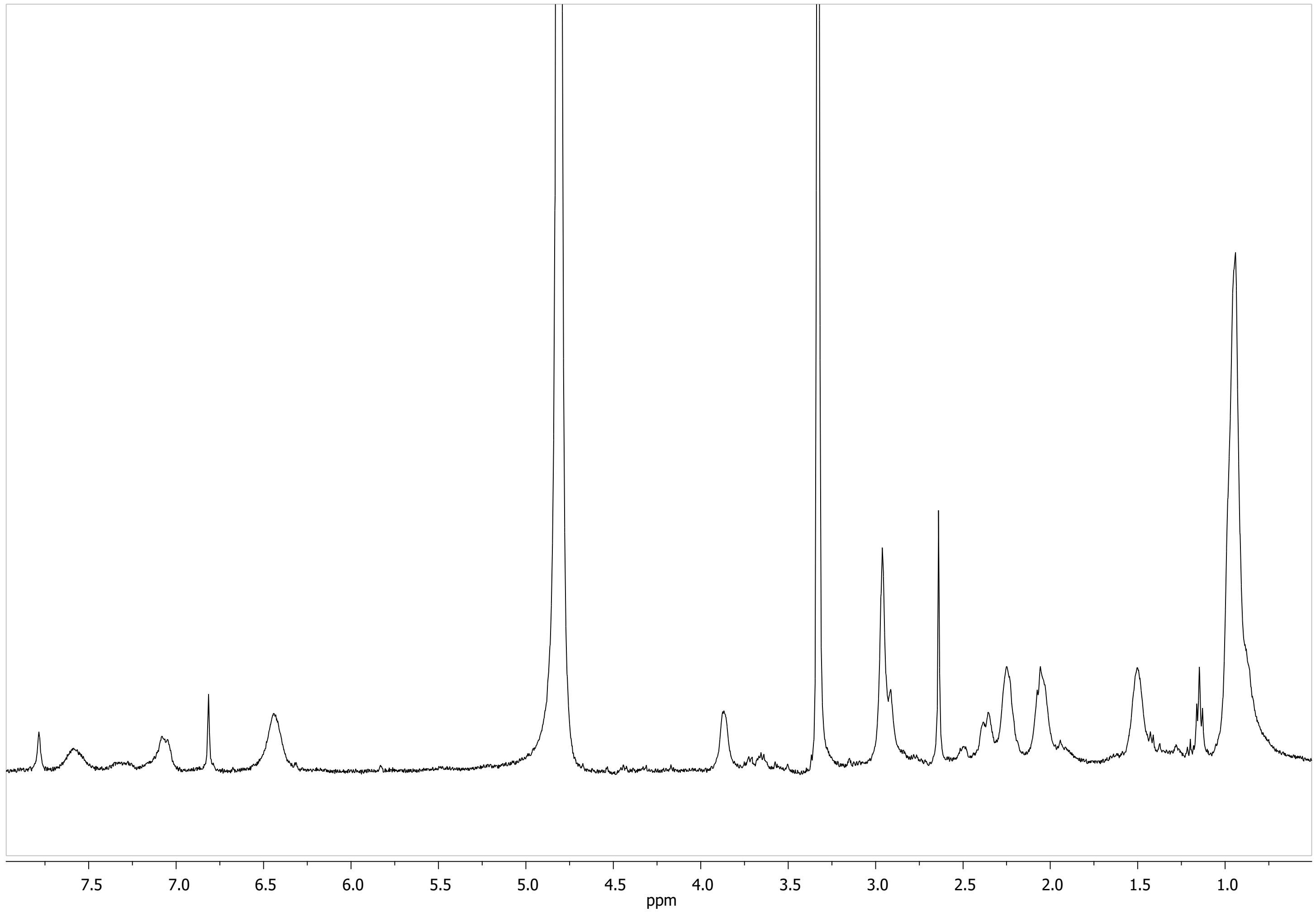

Figure S10. ^{1}H NMR spectrum of harzianic acid recorded in CD_3OD/D_2O (50:50 *w*/*w*) at 400 MHz

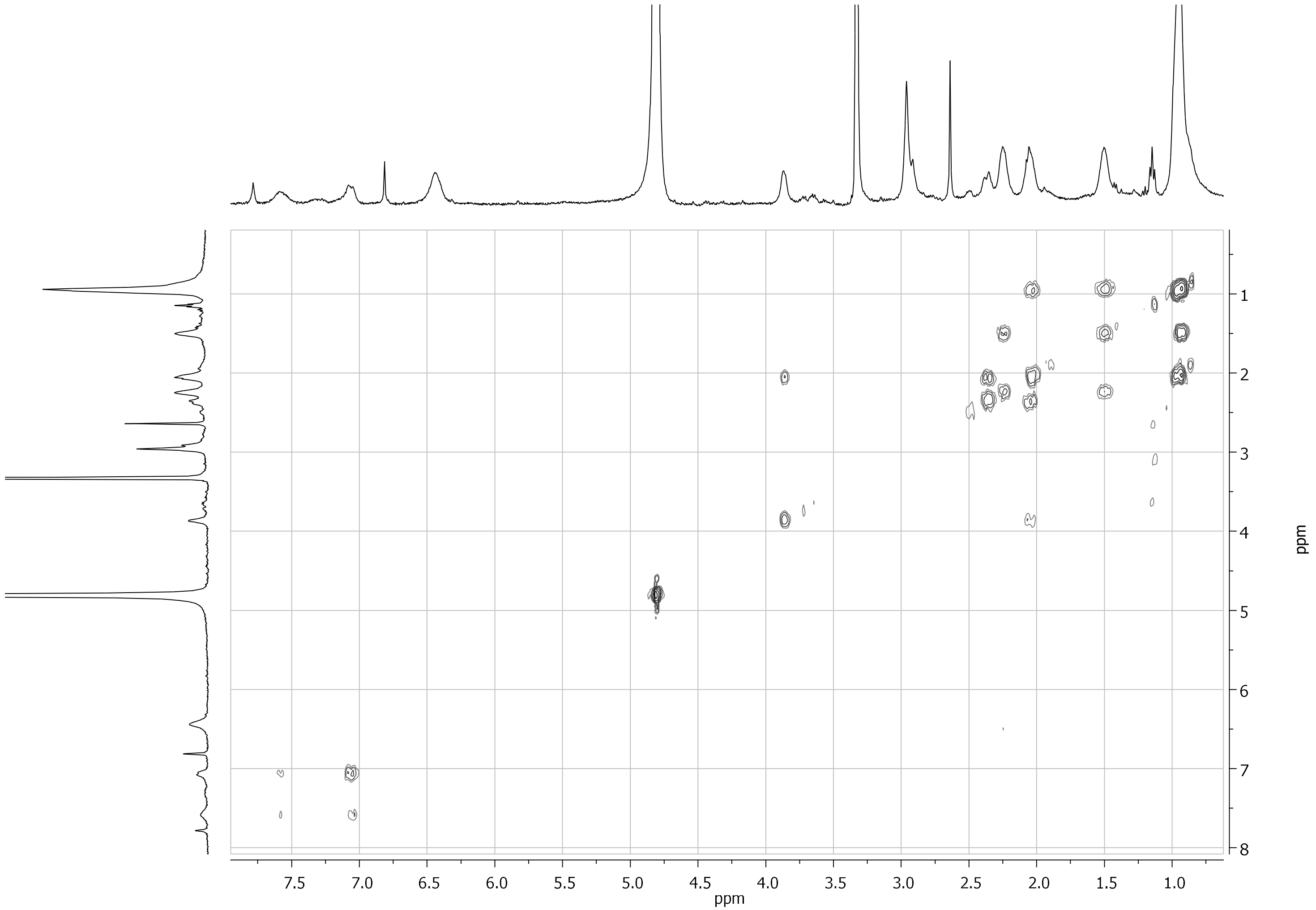

Figure S11. COSY spectrum of harzianic acid recorded in CD_3OD/D_2O (50:50 *w/w*) at 400 MHz

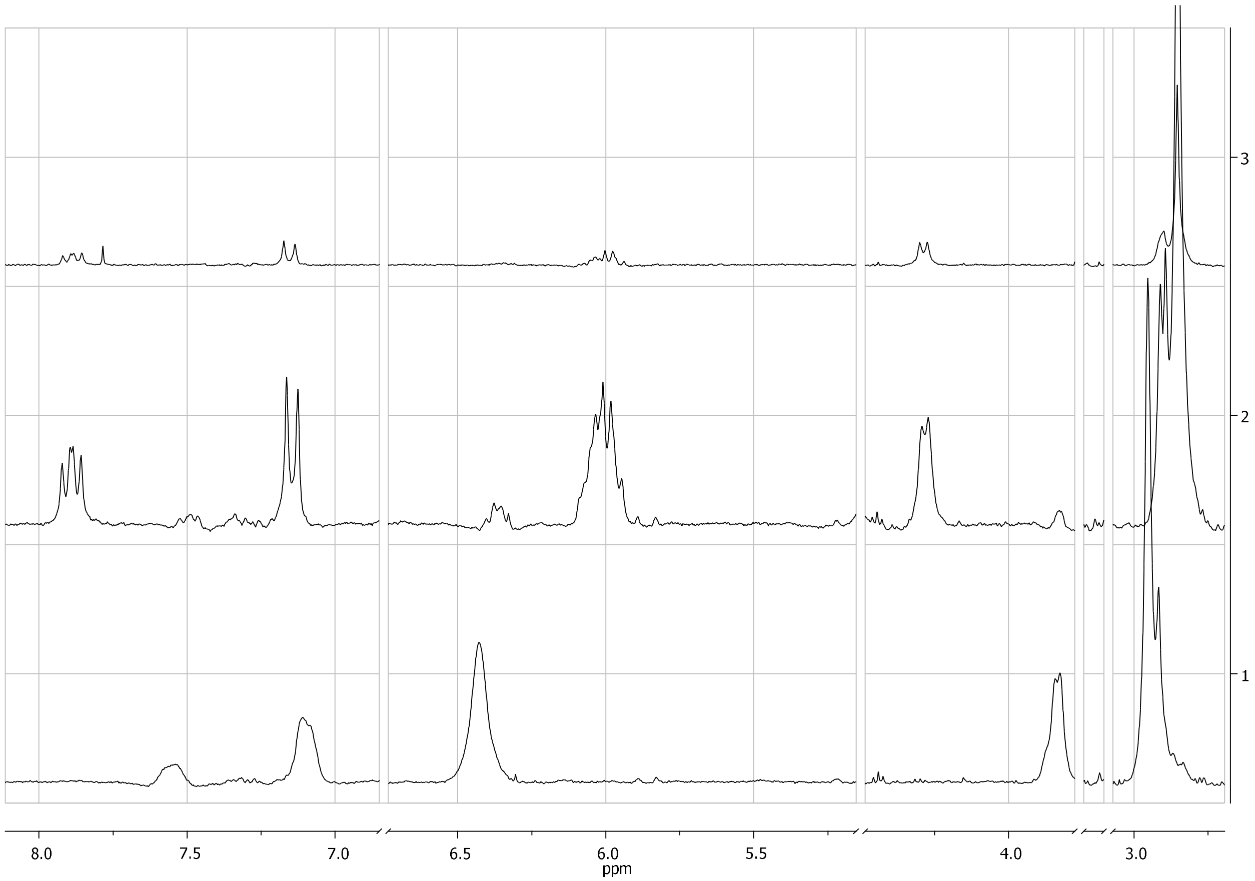

Figure S12. Detail of ^1H NMR spectra of harzianic acid (down), Zn^{2+}-HA (middle) and Mn^{2+}-HA (up) recorded in CD_3OD/D_2O (50:50 *w/w*) at 400 MHz. Solvent peak is removed.
